# Right-deviating prismatic adaptation reduces obsessions in a community sample

**DOI:** 10.3389/fpsyg.2022.1025379

**Published:** 2022-12-21

**Authors:** Barbara Magnani, Francesca Frassinetti, Christian Franceschini, Giancarlo Dimaggio, Alessandro Musetti

**Affiliations:** ^1^Department of Humanities, Social Sciences and Cultural Industries, University of Parma, Parma, Italy; ^2^Department of Psychology, University of Bologna, Bologna, Italy; ^3^Unit of Recovery and Functional Rehabilitation, Istituti Clinici Scientifici Maugeri IRCCS, Institute of Castel Goffredo, Mantua, Italy; ^4^Department of Medicine and Surgery, University of Parma, Parma, Italy; ^5^Center for Metacognitive Interpersonal Therapy, Rome, Italy

**Keywords:** obsessive-compulsive symptoms, spatial attention, prismatic adaptation, obsessions, patients with neglect

## Abstract

**Background and aims:**

Patients with obsessive-compulsive (OC) disorder are impaired in disengaging attention from negative valence stimuli and show an attentional bias toward the right space. This pattern in OC disorder is similar to the impaired disengagement of attention from stimuli in the ipsilesional space as a consequence of a right-hemispheric cerebral lesion in patients with neglect, suggesting a right hemispheric dysfunction in patients with OC disorder. The attentional impairment in patients with neglect is reduced by a visuomotor procedure, such as prismatic adaptation (PA) with right-deviating lenses. Thus, here, we explored whether right-deviating PA is also effective in reducing OC psychological symptoms.

**Methods:**

Participants with a high rate of OC symptoms completed self-report measures of such symptoms before and after right- or left-deviating PA.

**Results:**

Right-deviating PA, and not left-deviating PA, reduced OC symptoms more prominently on obsessions than compulsions.

**Conclusion:**

Results support the idea that right-deviating PA might be considered an effective technique to modulate OC symptoms. This has implications for theories about the underlying mechanisms of OC symptoms and the consideration of PA as a complementary procedure to psychological treatments.

## 1 Introduction

Different theories have tried to describe the physiological and psychological implications underlining obsessive-compulsive (OC) disorder. Some consider this disorder as stemming from alterations of the central nervous system, specifically in the cortico-striatal-thalamo-cortical network (Ahmari et al., 2013; Goodman et al., 2021). Others consider the symptoms related to OC disorder as a result of alterations in cognitive functions including memory, attention, and executive functions, such as decision-making and inhibition processes (Cameron et al., 2020). Cognitive theories, instead, assume that OC disorder emerges from how the person reacts to usually normal intrusive cognitions. The difference between psychologically healthy individuals and individuals with OC disorder is in the appraisal of such intrusive cognitions. OC disorder patients react to intrusive thoughts, such as the idea of harming someone, with a focus on harm and danger features. They consider these intrusive thoughts as evidence they may, for example, actually harm someone and they hold themselves accountable for the possible damage caused and focus on how to prevent danger (Salkovskis, 1985; Salkovskis et al., 1999). Other authors have found that obsessions and compulsions are triggered by attempts to avoid deontological guilt, that is, the idea of having broken moral rules (Mancini and Gangemi, 2015; Mancini, 2016).

An alternative perspective highlights the role of metacognitive processes exerted on intrusive cognition appraisal. In other words, once the intrusive cognition is appraised with a negative valence with respect to one’s own responsibility, individuals with OC disorder hold metacognitive beliefs aimed at controlling intrusive thoughts, such as “If I do not control a thought that worries me and it happens for real, I am to blame for the damage caused.” These higher-order judgments trigger repetitive thinking that is considered crucial for symptoms’ development and maintenance (Wells and Matthews, 1994; Myers and Wells, 2005).

Cognitive-behavioral therapies are formulated on these theoretical bases. Exposure and response prevention (ERP) protocol requires patients with OC disorder to face and manage conditions that provoke obsessions and to prevent compulsions they adopt to reduce the distress caused by their ideas (Reid et al., 2021, review). According to ERP, OC disorder improves by interrupting recursive behavioral processes that feed the disorder (Meyer, 1966). Other therapies focus on counteracting repetitive thinking (Fisher and Wells, 2008; Melchior et al., 2019). Nevertheless, results are far less than optimal (Maltby and Tolin, 2005; McKay et al., 2015), with significant rates of non-response or partial response, which calls for further investigation of mechanisms underlying OC symptoms.

One element that may foster our understanding of OC symptoms onset and maintenance may concern attentional impairment. Evidence has shown that patients with OC disorder display some aspects of brain functioning similar to patients with neglect syndrome. Neglect syndrome emerges after some cerebral lesions, caused for example by ischemia, most often in the right hemisphere. It consists of a deficit in orienting spatial attention toward the contralesional space (left) and an attentional bias toward the ipsilesional space (right), due to a deficit in disengaging attention from right-located stimuli (Posner et al., 1984; Driver and Mattingley, 1998; Sacher et al., 2004). As a result, when bisecting a line in its central point, patients with neglect systematically mark a point to the right of the true center (Marshall, 1998). When searching for stimuli on a sheet they perseverate in catching and signing the rightmost ones (Ferber and Karnath, 2001). Interestingly, neglect syndrome can also affect the mental imaginative space (Bisiach and Luzzatti, 1978; Rode et al., 2010) so that, when describing a mental space, patients with neglect only report the elements located on the right (Salvato et al., 2014).

In right-handers and the majority of left-handers, the right hemisphere is dominant for spatial attention, and it shifts attention toward the contralateral left space, while the left hemisphere shifts attention rightward (Kinsbourne, 1970; Corbetta and Shulman, 2011). Given the right hemispheric dominance for spatial attention, neurological healthy individuals explore and give more salience to the left space. Consistently, they systematically tend to bisect a line slightly toward the left (Brooks et al., 2014). Behavioral evidence shows that patients with OC disorder, similar to patients with neglect, bisect a line with a systematic rightward error (Rao et al., 2015). As such, Rao et al. (2015) interpreted the rightward error in line bisection in patients with OC disorder as a sign that the right hemisphere does not exert its dominant role in shifting attention to the left, resulting in an attentional bias to the right. Maril et al. (2007) studied patterns of spatial attention in patients with OC disorder adopting a Posner attentional paradigm (Posner, 1980). Here, participants need to detect and respond to stimuli in the left or right hemispace without moving their gaze from the center. Healthy participants responded faster to stimuli located on the left, whereas patients with OC disorder responded faster to stimuli located on the right. Interestingly, the greater the bias toward the right stimuli, the greater the severity of obsessions (Maril et al., 2007). Given the similarity between OC disorder and patients with neglect attentional performance, authors interpreted the result in patients with OC disorder as a sign of an imbalance between left and right hemispheric activity with a lower functioning of the right hemisphere compared to the left hemisphere. The relation between attentional performance and obsessions can be a sign that right hemisphere malfunctioning is one of the roots of OC symptoms.

Individuals with OC disorder have also difficulties in disengaging attention from stimuli with negative emotional valence (Morein-Zamir et al., 2013), such as fear- and disgust-inducing stimuli (Cisler and Olatunji, 2010). Crucially, there is evidence of an overlap between the processes involved in orienting attention to spatially located stimuli and salient emotional stimuli, and both are lateralized in the right hemisphere (Mäki-Marttunen et al., 2014; Spagna et al., 2020). These data suggest that disengagement deficit from negative emotional stimuli in patients with OC disorder can be another sign of a right hemisphere dysfunction.

The idea that OC and neglect symptoms share similar underlying mechanisms is also supported by dysfunctionality in the cortico-striatal-thalamo-cortical network subserved principally by dopaminergic regulation (Van Der Wee et al., 2004). Interestingly, both OC and patients with neglect show symptoms of improvement after dopamine-based interventions (Geminiani et al., 1998; Saxena et al., 1998; Gorgoraptis et al., 2012), and interventions usually adopted for neglect symptoms are shown to improve dopamine activity in healthy participants (Schintu et al., 2018).

**FIGURE 1:**
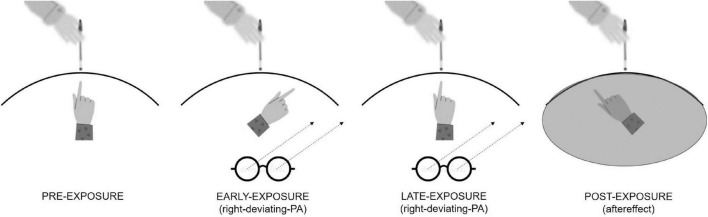
Graphical representation of right-deviating PA procedure inducing a leftward aftereffect. In the pre-exposure condition, participants do not wear prisms, and they can see their fingers so they exactly point to the visual target. In the early-exposure condition (i.e., first three trials), they wear prisms, and they mismatch the visual target with a pointing error toward the right. In the late exposure condition (i.e., last three trials), they adapt to prisms so they correctly point to the visual target despite wearing prisms. In the post-exposure condition, prisms are removed, and the participants cannot see their fingers. Here, they mismatch the visual target with a pointing error toward the left which corresponds to the aftereffect.

For all these reasons, there is room to speculate that treatments aimed at compensating for the attentional deficit in patients with neglect may reduce OC symptoms. One effective treatment for neglect is prismatic adaptation (PA) (Frassinetti et al., 2002; Serino et al., 2006). PA is a visuomotor procedure where participants point to a visual target wearing a pair of goggles (prisms), which shift the gaze toward a specific side of the visual space. The first pointing movements miss the visual target toward the same direction of the gaze shift. Since participants can see their fingers and spontaneously realize they are making an error, after some trials, they correct it (error reduction) and correctly point to the visual target. After prism removal, when participants are asked to point to the target, they miss it in the direction opposite to the prism deviation, showing the so-called aftereffect (Figure 1). The aftereffect is a sign of a spatial attention shift. Indeed, after right-deviating PA that induces a leftward shift of attention, patients with neglect bisect a line further to the left than before PA (Pisella et al., 2002); they also explore and report more elements in the left mental space (Rode et al., 2001) and disengage attention from right-located stimuli in favor of left-located stimuli (Shindler et al., 2009). The main hypothesis explaining right-deviating PA effects in patients with neglect is that PA “forces” the attentional systems toward stimuli located on the left, favoring a reorganization and a functional improvement of spatial attentional systems (Clarke and Crottaz-Herbette, 2016).

Our hypothesis is that right-deviating PA should stimulate attentional functioning in individuals with OC symptoms and reduce their symptoms. We recruited participants with OC symptoms and asked them to complete self-report questionnaires rating OC symptoms both before and after one session of right-deviating or left-deviating PA. We predicted that right-deviating PA, but not left-deviating PA, would reduce OC symptoms.

## 2 Materials and methods

### 2.1 Participants and procedure

Eighty-one right-handed university students aged 18-30 years with no history of neurological and psychiatric diseases and not being on medications completed the Obsessive-Compulsive Inventory-Revised (OCI-R, Italian version; Marchetti et al., 2010) after signing informed consent. Participants who scored above the clinical range (total score ≥ 84 percentile) were included in the study. We adopted as a stopping rule the sample numerosity (*n* = 26) indicated by the power analysis (conducted by G*Power 3.0.10, set with estimated minimum to medium effect size, f, of 0.25, with correlations among repeated measure, r, of 0.7 and powered at 85%).

The 26 participants were then randomly split into two groups. One group underwent a session of right-deviating PA (right-deviating PA group, 13 participants, 11 women, age mean = 23.5, *SD* = 3.2), and the other group underwent a session of left-deviating PA (left-deviating PA group, 13 participants, 8 women, age mean = 22.2, *SD* = 2.0). After PA, all participants completed again OCI-R. The interval between the OCI-R completion before and after PA was about 20 min. Both participants and experimenters were blinded to the purpose of the study.

The study was approved by the Ethics Board of the University of Parma (protocol number 0123743), and all procedures were performed in agreement with the Declaration of Helsinki.

### 2.2 Measures

Obsessive-compulsive inventory-revised (OCI-R): We adopted this self-report measure assessing OC symptoms for screening participants because of its sensitivity to change in reported symptoms following experimental manipulations (Marchetti et al., 2010). It is composed of 18 items, 3 for each of the 6 scales corresponding to OC symptoms categories, namely, hoarding, checking, ordering, mental neutralizing, washing, and obsessing. Participants respond on a Likert scale from 0 to 4: the higher the score, the greater the symptoms. The test–retest reliability for a monthly interval ranges from 0.76 to 0.99 (Marchetti et al., 2010).

PA procedure: Participants sat at a table in front of a box (height = 30 cm, depth = 34 cm at the center and 18 cm at the periphery, width = 72 cm) that was open on the side facing the participant and on the opposite side, facing the experimenter. The experimenter placed a visual target (a pen) at the distal edge of the top surface of the box, in one of three possible positions (randomly determined on each trial): central (0°), 21° to the left, and 21° to the right. For each target placement, participants performed the pointing task. It consisted in keeping their right hand at the level of the sternum and then pointing toward the pen using the index finger of the same hand. The experimenter recorded the end position of the participant’s pointing direction. In invisible pointing trials, the arm was totally covered by a black sheet, and the participants did not see any part of the trajectory of the arm. In visible pointing trials, the arm was covered only in the proximal part, and the participants could see the last third of the trajectory of the pointing movement. The conditions of the pointing task were structured as follows: pre-exposure condition: 60 trials, 30 in visible pointing and 30 in invisible pointing; exposure condition: 90 trials in visible pointing, participants wore prismatic lenses that induced a 20° shift of the visual field to the left or the right; and post-exposure condition: 30 trials in invisible pointing, immediately after removal of the prisms. In the latter condition, the pointing movement should shift in the opposite direction of the visual shift induced by prisms, called aftereffect.

## 3 Analysis

All analyses have been performed using SPSS version 29 software. First, we ensured that the two groups were balanced for the level of OC symptoms before PA. We ran an ANOVA on the OCI-R raw scores obtained before PA with group (right-deviating PA group and left-deviating PA group) as a between-factor and scales (hoarding, checking, ordering, mental neutralizing, washing, obsessing, and total) as a within-subjects variable.

Second, we ensured that participants showed the effects attesting to the efficacy of the PA procedure: *error reduction* and *aftereffect.* To verify that participants showed *error reduction*, we ran an ANOVA on the mean displacement (expressed as degrees of visual angle—°) of the participants’ visible pointing, with group (right-deviating PA group and left-deviating PA group) as a between-factor variable and condition (pre-exposure, first three trials of exposure, and last three trials of exposure condition) as a within-subjects variable. Successively, to be sure that each group obtained the expected error reduction, for each prismatic deviation, we ran an ANOVA on the mean pointing displacement of the participants’ visible pointing with condition (pre-exposure, first three trials of the exposure, and last three trials of exposure condition) as a within-subjects variable. To verify whether participants showed *aftereffect*, we ran an ANOVA on the mean displacement (expressed as degrees of visual angle) of the participants’ invisible pointing, with group (right-deviating PA group and left-deviating PA group) as a between-factor variable and condition (pre-exposure condition and post-exposure condition) as a within-subjects variable. To assess that each group obtained the expected aftereffect, for each prismatic deviation, we ran a paired sample *t*-test (two-tailed) to compare the mean pointing displacement of the participants’ invisible pointing in the post-exposure vs. pre-exposure condition.

Third, we investigated the effects of PA on OCI-R scores. To compare the effects of prismatic deviation on OC symptoms, we ran a repeated measure ANOVA on the mean of OCI-R raw scores with group (right-deviating PA group and left-deviating PA group) as a between-factor variable and session (before-PA vs. after-PA) and scales (hoarding, checking, ordering, mental neutralizing, washing, obsessing, and total) as within-subjects variables. Then, we ran paired sample *t*-tests (two-tailed) to compare after PA vs. before PA scores on OCI-R *singular scales*. Moreover, for each prismatic deviation, we ran an additional ANOVA with the session (before-PA vs. after-PA) and items as within-subjects variables and paired sample *t*-tests (two-tailed) to compare after PA vs. before PA scores on OCI-R *singular items*.

Finally, we explored whether changes in scores found after vs before PA were associated with PA efficacy, measured by error reduction (first three trials minus last three trials of visible pointing exposure condition means) and aftereffect (post-exposure minus invisible pointing pre-exposure condition means). We ran a Pearson correlation analysis, for each prismatic deviation, with error reduction, aftereffect, and OCI-R score reduction (after PA minus before PA) of *scales* and *items* as variables. Bonferroni correction was applied on significant p-values.

For all significant F, we reported the effect size as partial eta square, and for all significant *t*, we reported the effect size as Cohen’s *d* estimated point.

## 4 Results

### 4.1 Levels of OC symptoms before PA

The two groups were balanced for the level of OC symptoms before PA since the interaction group x scales was not significant (p = 0.64).

### 4.2 Efficacy of PA procedure

Both groups showed the expected error reduction and aftereffect.

#### 4.2.1 Error reduction

**FIGURE 2:**
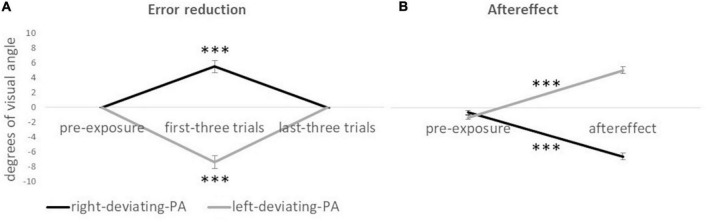
Mean displacement (in degrees of visual angle) of pointing responses for the right-deviating PA and the left-deviating PA groups. Panel **(A)** shows error reduction values. ^***^Symbol (*p* < 0.001) indicates the effect of condition (visible pointing pre-exposure condition, first three trials of the exposure condition, and last three trials of exposure condition) for the ANOVA on each prismatic deviation. Panel **(B)** shows aftereffect values. ^***^Symbol (*p* < 0.001) indicates the difference between the invisible pointing pre-exposure condition and aftereffect condition for the paired sample *t*-test (two-tailed) on each prismatic deviation. Negative values indicate a leftward pointing displacement, and positive values indicate a rightward pointing displacement with respect to the real location of the target. Error bars indicate standard errors of means.

The interaction group x condition was significant [*F*(2,23) = 56.77; *p* < 0.001; ηp2 = 0.832] indicating a difference between right-deviating and left-deviating PA groups in the first three trials and not in the pre-exposure (visible pointing) and the last three trials (Figure 2A for all values). For the right-deviating PA, the ANOVA showed that the condition was significant [*F*(2,11) = 22.95; *p* < 0.001; ηp2 = 0.807]: the rightward pointing displacement in the first three trials (5.49°) was bigger than that in the pre-exposure condition (-0.01°) and the last three trials (0.01°). Specularly, for the left-deviating PA, the ANOVA showed that the condition was significant [*F*(2,11) = 31.80; *p* < 0.001; ηp2 = 0.853]: the leftward pointing displacement in the first three trials (−7.34°) was bigger than that in the pre-exposure condition (−0.01°) and the last three trials (−0.04°, Figure 2A for standard errors). The absence of pointing displacement (around 0°) in the pre-exposure condition and last trials of the exposure condition is an index of adaptation to prismatic lenses.

#### 4.2.2 Aftereffect

The interaction group x condition was significant [*F*(1,24) = 495.32; *p* < 0.001; ηp2 = 0.954]: the two groups differed in the aftereffect condition but not in the invisible pointing pre-exposure condition (Figure 2B for all values). For the right-deviating PA, the paired sample *t*-test showed a significant effect *t*(12) = 15.61; *p* < 0.001; *d* = 4.33, indicating that the leftward pointing displacement was bigger after PA (−6.37°) than before PA (−0.57°). Specularly, for the left-deviating PA, the paired sample *t*-test showed a significant effect *t*(12) = 15.86; *p* < 0.001; *d* = −4.40, indicating that the rightward pointing displacement was bigger after PA (4.99°) than before PA (-1.22°, Figure 2B).

### 4.3 Effects of PA on OCI-R scores

**FIGURE 3:**
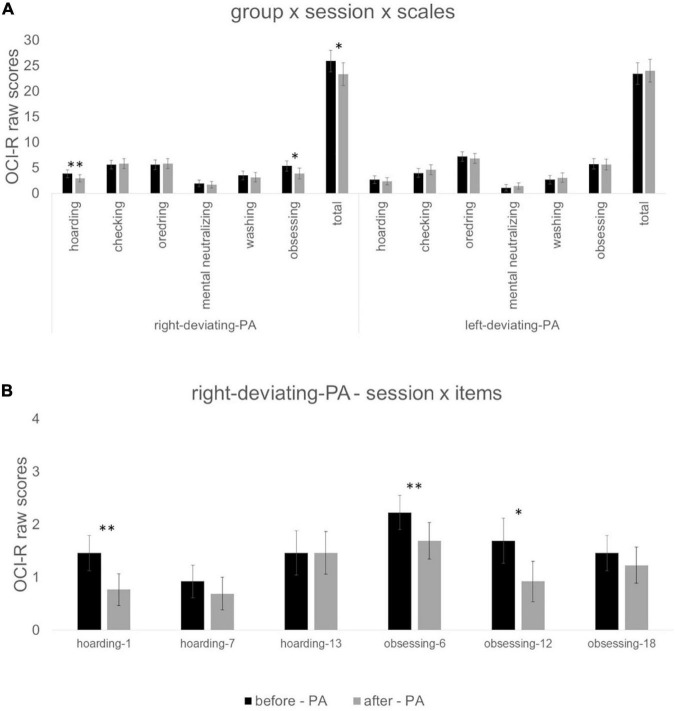
Panel **(A)** indicates values of the interaction group (right-deviating-PA and left-deviating PA) x session (before PA vs. after PA) x scales (hoarding, checking, ordering, mental neutralizing, washing, obsessing, and total). Panel **(B)** indicates values of the interaction: session (before PA vs. after PA) x items (1, 7, 13, 6, 12, 18) for the right-deviating PA group. *Symbol indicates *p* < 0.05 and ^**^symbol indicates *p* < 0.01 of paired sample *t*-tests. Error bars indicate standard errors of means.

As regards the effect of PA deviation on OCI-R scores, the effect of group x session x scales was significant [F(6,19) = 2.67; *p* < 0.05; ηp2 = 458]. For the right-deviating PA group, *t*-tests showed a significant reduction in scores after vs before PA for the scale hoarding *t*(12) = 3.21, *p* < 0.01, *d* = 0.89; obsessing *t*(12) = 2.80, *p* < 0.05, *d* = 0.76; and total *t*(12) = 2.43, *p* < 0.05, *d* = 0.67. For the left-deviating PA group, *t*-tests were not significant (*p* > 0.24 for all comparisons—Figure 3A for all values).

For the right-deviating PA group, the ANOVA with session and items of hoarding and obsessing scales (items 1, 7, 13, 6, 12, 18) showed a significant session x items [*F*(1,5) = 2.61; *p* < 0.05; ηp2 = 0.179] interaction. Paired sample *t*-tests showed a reduction in scores after vs. before PA for item 1 of hoarding scale *t*(12) = 3.32, *p* < 0.01, *d* = 0.92; item 6 obsessing scale *t*(12) = 3.74, *p* < 0.01, *d* = 1.04; and item 12 of obsessing scale *t*(12) = 2.25, *p* < 0.05, *d* = 0.62 (Figure 3B for all values).

### 4.4 Correlational analysis

The Pearson correlation analysis for the right-deviating PA on error reduction, aftereffect, and score reduction in hoarding scale, obsessing scale, item 1, item 6, and item 12 revealed a negative correlation between item 6 (“I find it difficult to control my thoughts.”) and error reduction (r = -0.714, R^2^ = 0.51, p < 0.01, Bonferroni correction p*7 < 0.05): the higher the error reduction the lower the score on item 6 after vs. before PA. This indicates that the more participants correct the pointing error, the lower the effort that participants perceive in controlling their thoughts after vs. before PA.

## 5 Discussion

We investigated whether PA, a technique that shifts spatial attention proven effective in alleviating attentional deficit in patients with neglect, reduces OC symptoms. We administered PA to 26 students with clinically significant levels of OC symptoms as assessed with the OCI-R. The results were consistent with our prediction: right-deviating PA, but not left-deviating PA, reduces OC symptoms. We also found that right-deviating PA is mainly effective on the OCI-R measures of obsessions rather than compulsions.

What are the possible explanations for our results? It seems that the leftward aftereffect produced by right-deviating lenses restores attentional functions, which are tied to the right hemisphere. In OC disorder, the difficulty to disengage attention from the right (Rao et al., 2015) and negative valence stimuli (Morein-Zamir et al., 2013) are both associated with a dysfunction of the right hemisphere. Accordingly, there is evidence that not only spatial attention but also emotional valence appraisal relies upon right hemisphere circuits (Hartikainen, 2021, review). Since attentional systems in the human brain have evolved to allocate attention as quickly as possible to stimuli important for survival, attentional resources located in the right hemisphere are captured by negative valence stimuli. Stimuli with negative emotional valence, e.g., threat stimuli, compete for spatial attentional resources in the right hemisphere (Hartikainen et al., 2000). Consistently, a reduction of attention to stimuli located on the left (processed by the right hemisphere) when they are preceded by emotionally unpleasant stimuli has been demonstrated (Hartikainen et al., 2007). As a consequence, a dysfunctional right hemisphere, with poor resources, tends to prioritize negative stimuli instead of positive ones (Hartikainen, 2021). This hyperattention to the negative valence of stimuli may support the establishment of obsessive mechanisms. Once the negative valence is implicitly attributed, an increase in arousal informs high-order cognitive processes that “something is wrong.” Then, individuals with OC symptoms are triggered toward obsessions about the real presence of danger or, alternatively, about their own possible responsibility to prevent it (Mancini and Gangemi, 2015). According to our results, it is possible that in individuals with OC symptoms, a restoration of the right hemisphere by right-deviating PA reduces hyperattention to negative valence stimuli. Reduced attention to negative stimuli could defuse obsessions establishment.

In line with this interpretation, stimulation of the left hemisphere induced by left-deviating PA (Magnani et al., 2014) is ineffective on OC symptoms, as self-reported OC symptoms do not change after left-deviating PA compared to before. However, it can be argued that left-deviating PA should exasperate the hyperattention to negative valence stimuli leading to a worsening of obsessions. Looking at our results, the lack of effect of the left-deviating PA can be interpreted as a ceiling effect of hyperattention to negative stimuli in individuals with OC symptoms. If this was true, left-deviating PA in individuals without OC symptoms should induce obsessions, but our experiment did not investigate this possibility.

A somewhat unexpected finding was the lack of the right-deviating PA effect on compulsions. This is possibly due to the fact that compulsions are sustained by behavioral mechanisms that may need more time and wilful effort to be interrupted, such as during exposure and response prevention protocol (ERP). Alternatively, just one trial of adaptation to right-deviating prisms in our laboratory condition was insufficient to counteract behavior habits, while repeated applications may display such an effect. It is also worth noting that we used self-report measures of OC symptoms instead of behavioral paradigms, which would have been more sensitive to eventual changes in compulsions. Finally, since we studied a non-clinical sample, it remains to be investigated whether right-deviating PA reduces compulsions in a clinical sample suffering from full-blown OC disorder.

A reduction of OC symptoms, after right-deviating PA stimulating the right hemisphere, is also consistent with neurophysiological evidence. An increment of the resting motor threshold of the right hemisphere after repetitive transcranial magnetic stimulation (rTMS) treatment was associated with a reduction of OC symptoms (Mantovani et al., 2006). Ma et al. (2021) scanned OC disorder patients with resting-state functional magnetic resonance (rs-fMRI) before and after cognitive-coping therapy. The increase of resting state activity (i.e., spontaneous neural activity) in the right hemisphere after cognitive-coping therapy was associated with the reduction of OC symptoms severity. Mosley et al. (2021) studied the efficacy of a deep brain stimulation (DBS) protocol combined with cognitive-behavioral therapy in patients with OC disorder. The optimal response to DBS was associated with the structural connectivity of the stimulated tract in the right hemisphere. Moreover, the connectivity of a right frontoparietal network, the one responsible for spatial attention, was generally associated with the reduction of symptoms (Mosley et al., 2021).

Despite the promising results, we acknowledge that our study has some limitations. The sample was small and women were prevalent. Moreover, we did not control for the effects of a sham condition, for example, PA with neutral lenses. Finally, this was a non-clinical sample so results cannot be generalized to patients diagnosed with OC disorder.

In conclusion, the present study puts forward the idea that right-deviating PA in individuals with OC symptoms may reduce obsessions. These findings open the road for a larger study aimed at exploring if PA has an effect on obsessions or compulsions or both in patients diagnosed with OC disorder, and it can be considered an add-on aimed at increasing the effectiveness of existing treatments.

## Data availability statement

The raw data supporting the conclusions of this article will be made available by the authors to interested researchers.

## Ethics statement

The study involving human participants was reviewed and approved by Ethics Committee of the University of Parma - protocol number: 0123743. The patients/participants provided their written informed consent to participate in this study.

## Author contributions

BM was salient in conceptualizing and conducting the experiment and in writing the manuscript. AM was salient in discussing results and in managing collaborations among members of the research team. CF was precious in discussing results especially under a statistical perspective. FF and GD were crucial in conceptualizing and supervisioning the research especially at a theoretical level. All authors contributed to the manuscript in a meaningful way.
